# Relating genomic characteristics to environmental preferences and ubiquity in different microbial taxa

**DOI:** 10.1186/s12864-017-3888-y

**Published:** 2017-06-29

**Authors:** Marta Cobo-Simón, Javier Tamames

**Affiliations:** Systems Biology Programme, Centro Nacional de Biotecnología (CNB-CSIC), C/Darwin 3, 28049 Madrid, Spain

**Keywords:** Microorganisms, High-throughput sequencing, Functional capabilities, Genomics, Co-occurrences

## Abstract

**Background:**

Despite the important role that microorganisms play in environmental processes, the low percentage of cultured microbes (5%) has limited, until now, our knowledge of their ecological strategies. However, the development of high-throughput sequencing has generated a huge amount of genomic and metagenomic data without the need of culturing that can be used to study ecological questions. This study aims to estimate the functional capabilities, genomic sizes and 16S copy number of different taxa in relation to their ubiquity and their environmental preferences.

**Results:**

To achieve this goal, we compiled data regarding the presence of each prokaryotic genera in diverse environments. Then, genomic characteristics such as genome size, 16S rRNA gene copy number, and functional content of the genomes were related to their ubiquity and different environmental preferences of the corresponding taxa. The results showed clear correlations between genomic characteristics and environmental conditions.

**Conclusions:**

Ubiquity and adaptation were linked to genome size, while 16S copy number was not directly related to ubiquity. We observed that different combinations of these two characteristics delineate the different environments. Besides, the analysis of functional classes showed some clear signatures linked to particular environments.

**Electronic supplementary material:**

The online version of this article (doi:10.1186/s12864-017-3888-y) contains supplementary material, which is available to authorized users.

## Background

Many microbes remain unculturable despite recent progress in cultivation techniques [1–7]. The development of high-throughput sequencing has provided a way to circumvent this limitation, producing a very large amount of genomic data that informs of the possible functions and capabilities of the targeted microorganisms. However, despite this huge amount of complex data [8], our knowledge of the ecological strategies that underlie microbial diversity remains limited [9–11].

Establishing the ecological principles that shape the distribution of microbial taxa is a major challenge [12–14]. The importance of environmental factors on such distribution has been demonstrated, either considering only biotic factors [13, 14], or also including interactions among different taxa and abiotic factors [15]. Besides, the analysis of genomic data from an ecological point of view can provide a more comprehensive understanding of the structure and functioning of microbial communities [16, 17].

Several studies have analyzed the interrelationships among species considering the co-occurrences of different taxa in the same environments [12, 14, 18]. However, although thousands of fully sequenced genomes are available and some ecological traits have been investigated using metagenomic data [11, 15, 19, 20], these studies have mostly focused on specific environments. Recently, we have described the global relationships between genomic content, phylogenetic distance, and environmental preferences [21]. Following that approach, the main objective of this study is to find patterns between genomic characteristics of different prokaryotic taxa and both their ubiquity and their environmental preferences.

To this end, we addressed the relationships between these environment preferences and both genome size and the copy number of the 16S rRNA gene. We chose these genomic characteristics because previous studies related genome size to environmental versatility [22] and 16S gene copy number to the potential growing rate [23]. In addition, we also analyzed the functional capabilities of each genus in comparison with its ecological strategies and environmental preferences. Since our study is focused on environmental versatility and growing rate, we will obtain insight on the ecological strategies to study linked to these traits, namely: (i) oligotrophic/copiotrophic microorganisms (growth rate) and (ii) generalist/specialist microorganisms (environmental versatility/ubiquity).

## Methods

Genomic data were obtained for 2837 complete prokaryotic genomes with COGs (Clusters of Orthologous Groups) annotations [24, 25], taken from the National Center of Biotechnology Information (NCBI) [26]. As some genomes are scarcely annotated, we only considered these genomes in which the normalized ratio of annotated genes versus the total of genes fell within one standard deviation of the mean ($\overline{x}$ = 71.5%, SD = 15.7). This resulted in 1420 genomes. We also calculated the genome size, the copy number of 16S rRNA gene and the number of different copies of it (that is, non-identical 16S rRNA genes) for these genomes. Next, we created a functional profile of each genome by counting the abundance of individual COGs, that are classified in 18 individual functional classes. We normalized the number of ORFs (Open Reading Frames) and COGs in each functional class by dividing by the total number of ORFs and COGs, respectively. For validating purposes, we also created functional profiles based on KEGG (Kyoto Encyclopedia of Genes and Genomes) annotations [27].

We obtained the environmental data from the microDB data base (formerly envDB, http://botero.cnb.csic.es/envDB) [28], following the procedure in [29]. The database comprises more than 20,000 environmental samples and their associated 16S rRNA sequences, with each sample classified in a unique environment. By taxonomically classifying the 16S sequences in these samples, it is possible to know which taxa are present in which samples and, consequently, in the corresponding environments. We chose the genus level as taxonomic working unit because it provides a good balance between a high number of taxa and not too sparse observations for them. Also, many 16S sequences in microDB are partial and do not allow classification to ranks below genera.

In order to obtain the number of environments where each genus was found, we computed a table of the observed frequency of genera in environments (Additional file 1), excluding instances in which a genus was observed in just one sample of a particular environment. Using these frequencies, we tested the measure of the association genus-environment using Fisher’s exact tests, adjusting the resulting *p*-values using the Benjamini and Hochberg FDR correction [30]. We considered that a taxon was linked to an environment when the adjusted *p*-value was lower than 0.01. To assess that these significances were not random observations, we computed 100 random frequency tables and applied the same procedure to them. No significant associations were detected between genera and environments using these random matrices (*p*-value = 1). The number of genomes in each environment, and the number of environments linked to each genome, can be found in Additional file 2.

Since each genus can contain different species/strains, we averaged the values of the genome size, copy number of 16S rRNA gene, the number of different 16S rRNA genes and the COGs/KEGGs functional profile for the corresponding genomes.

Finally, to link COG presence with ubiquity, we calculated the average presence of each COG across all the genomes of a genus (thus obtaining a value between zero and one), and related that measure with the ubiquity of the genus.

All statistical tests were done using the software package R [31].

## Results

### Relationships of 16S copy number, number of different 16S genes, genome size and functional profile for every genus with ubiquity and environmental preferences

**Fig. 1:**
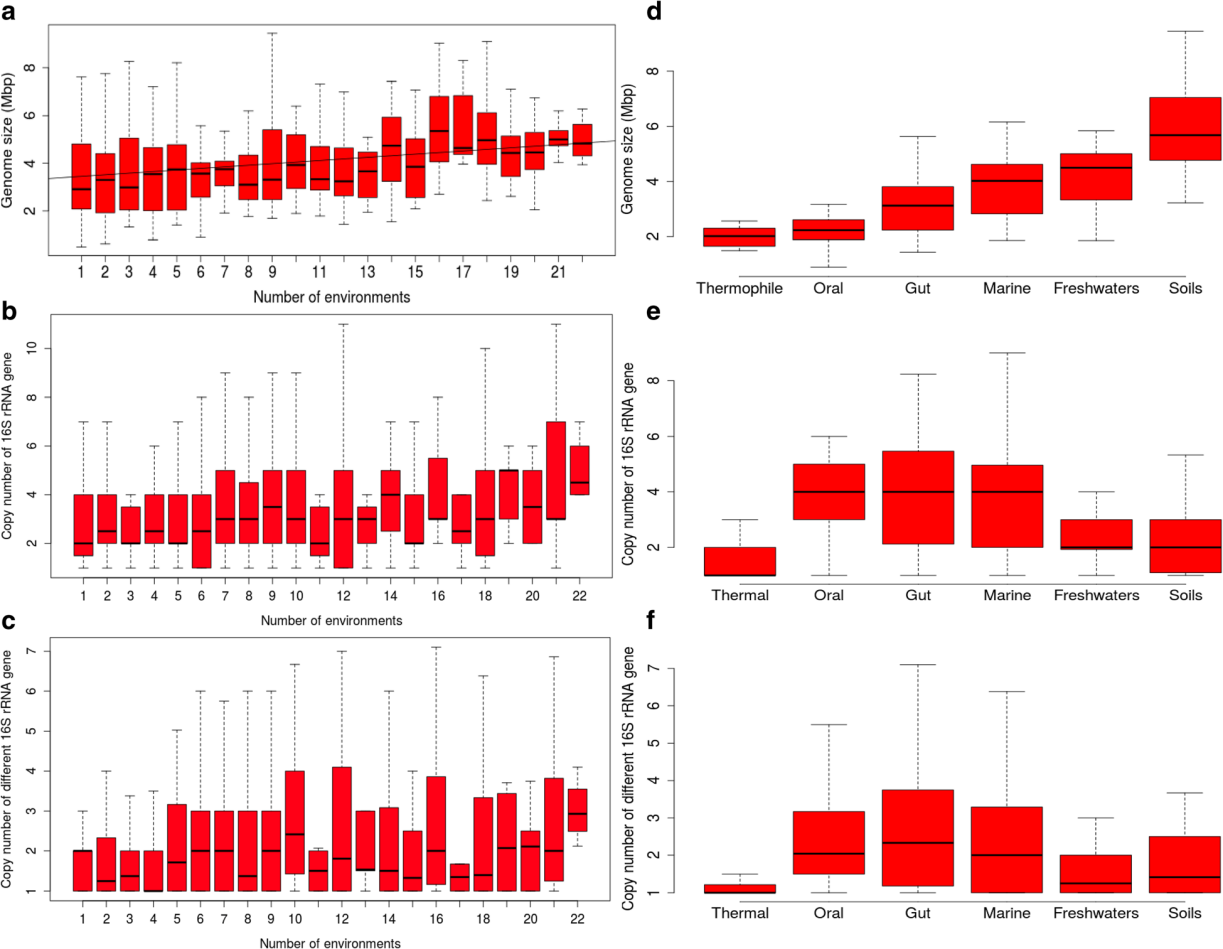
Relationship between the number of environments for each genus and the genome size (**a**), copy number of 16S rRNA gene (**b**) and number of different 16S rRNA genes for the genomes belonging to these genera (**c**). The same comparison was made related to the type of environment (**d**, **e** and **f**, respectively). The number of used genomes is provided above each group

Figure 1 and Additional file 3 show the relationships between genome size, 16S rRNA copy number and number of different 16S rDNA genes with the ubiquity of their corresponding genomes (Fig. 1a-c) and the particular environments associated with them (Fig. 1d-f, Additional file 3). Genome size increased with ubiquity (Fig. 1a), which was confirmed by a linear regression (R^2^ = 0.04, *p*-value <0.01) and a Kruskal-Wallis significance test (*p*-value <0.01). In contrast, the 16S rRNA gene copy number (Fig. 1b) did not vary according to the ubiquity (Kruskal-Wallis *p*-value >0.01). The number of different 16S rRNA genes did not vary either according to the ubiquity (Kruskal-Wallis *p*-value >0.01) (Fig. 1c).

**Fig. 2:**
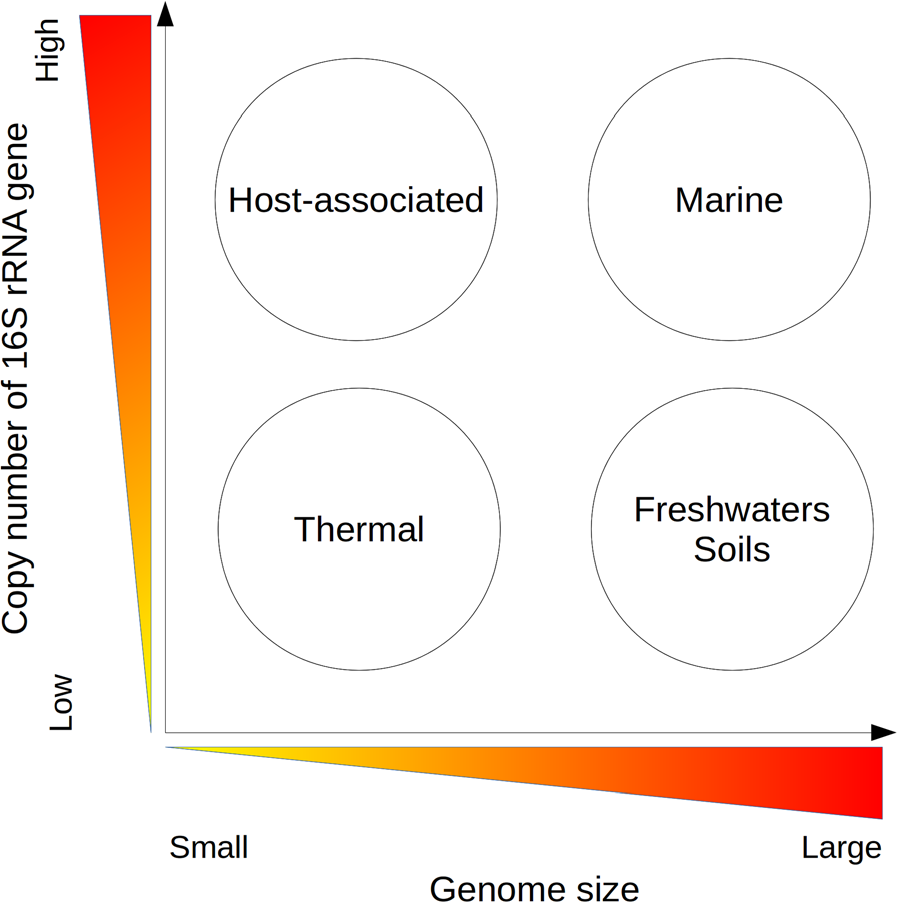
Trends for genome size and copy number of the 16S rRNA gene in different environments

Microorganisms living in different environments showed different trends regarding genome size (Kruskal-Wallis *p*-value <0.01) and 16S rRNA copy number (*p*-value < 0.01) (Fig. 1d-f). The genera occurring in host-associated environments (gut and oral) showed both high copy number of 16S rRNA genes and small genome size (Fig. 1d-e, Additional file 3A). Thus, according to the results of Mann-Whitney statistical test, both environments did not present significant differences in copy number and genome size (*p*-value >0.01) but they were significantly different for genome size and copy number of 16S rRNA gene from the rest of environments (*p*-value <0.01) . Genera present in soils (Fig. 1d-e, Additional file 3B) showed significantly the largest genome sizes of the data set (Mann-Whitney *p*-value <0.01) but no statistical differences in 16S rRNA copy number with the other environments (*p*-value >0.01) (Fig. 1d-e, Additional file 3C). Thermophilic bacteria inhabiting springs and submarine vents showed both significantly low 16S rRNA copy number and small genome size (the smallest genomes) (Mann-Whitney test *p*-value <0.01) (Fig. 1d-e, Additional file 3D). Marine and freshwaters microorganisms did not present significant differences in genome size and 16S rRNA gene copy number (Additional file 3), but although both tended to have large genomes, freshwaters presented a slightly lower copy number of 16SrRNA gene than marine genera (Fig. 1d-e), being closer to soils microorganisms.. The results of the different 16S rRNA genes showed the same tendency as 16S copy number (Fig. 1c, f). According to these results, we can extract some general trends for environmental microorganisms, summarized in Fig. 2.

### Relationships between gene/functional class content, ubiquity and environmental preferences

Next, we studied the distribution of functions in the genomes linked to the diverse environments, to gain insight on: 1) the adaptations to ubiquity, and 2) the adaptations to different environments.

**Fig. 3:**
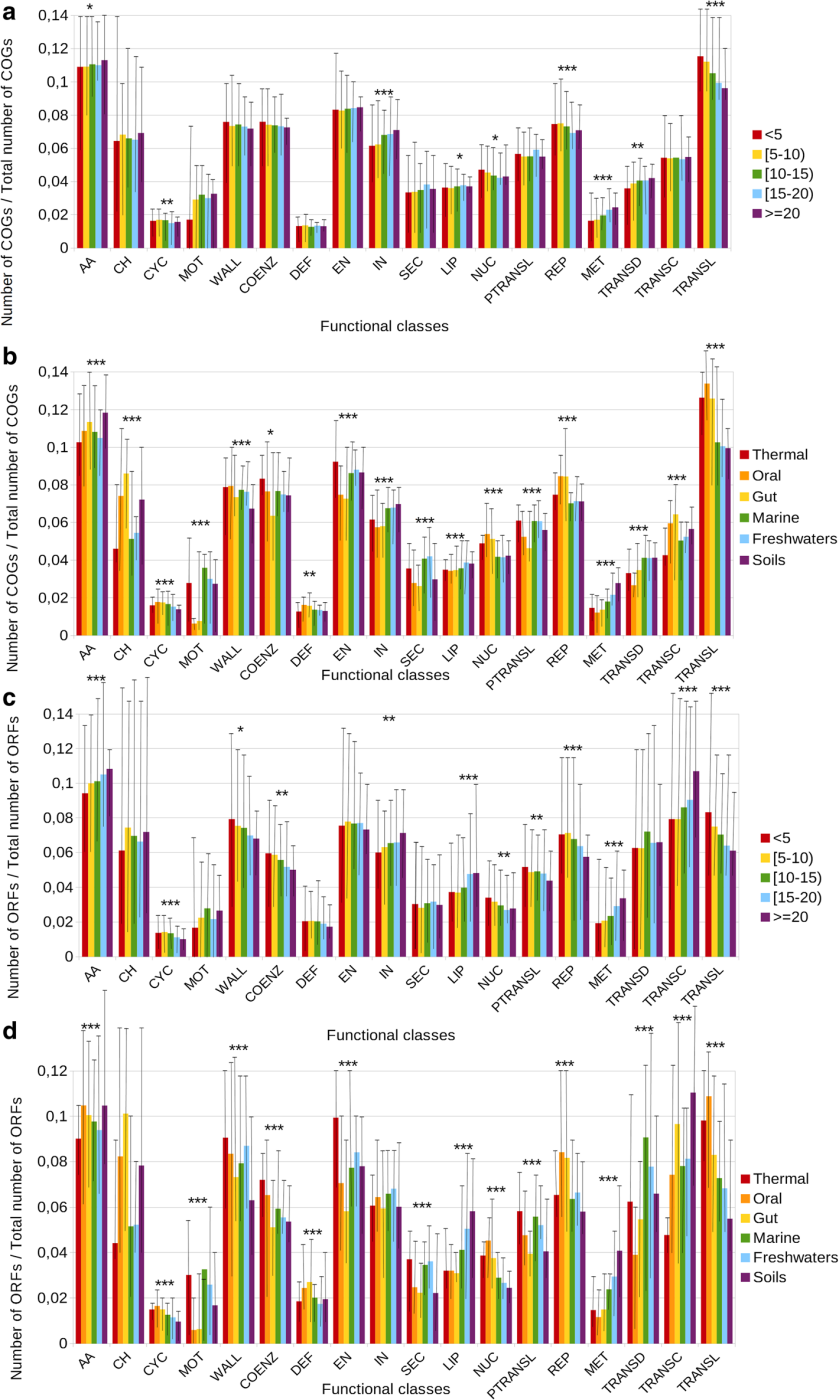
Fraction of distinct COGs (**a** and **b**) and ORFs (**c** and **d**) dedicated to different functional classes regarding their ubiquity (**a** and **c**), and the preferred environment for each genus (**b** and **d**). The *error bars* represent the range of the data

We calculated the ratio of different COGs (Fig. 3a and b) and ORFs (Fig. 3c and d) that each genus devotes to each functional class, grouping the genera according to either the number of environments in which they were found (Fig. 3a and c), or by their preferred environments (Fig. 3b and d). The ratio of COGs will inform on the number of different protein families for a particular functional class, whereas the ratio of ORFs will tell us of the number of total genes for it.

To verify that these trends hold when using a different functional classification, we repeated the analyses using KEGG database (Additional file 4). The results indicate that equivalent functional classes follow the same trends almost entirely.

First we focused on studying the ratio of different COGs. For most functional classes, we did not find any correlation with the ubiquity of the corresponding bacteria (Fig. 3a). Only the classes of inorganic ion transport and metabolism and secondary metabolism increased their share in ubiquitous taxa (*p*-value Kruskal-Wallis test <0.01, linear regression *p*-value <0.01, R^2^ = 0.61 and 0.68 respectively). In contrast, translation, ribosomal structure and biogenesis significantly decreased with ubiquity (*p*-value Kruskal-Wallis test <0.01, linear regression *p*-value <0.01, R^2^ = 0.61). When using the KEGG database, lipid metabolism, metabolism of other aminoacids and xenobiotics metabolism increased their ratio in ubiquitous taxa (*p*-value Kruskal-Wallis test <0.01, *p*-value linear regression <0.01, R^2^ = 0.41, 0.36 and 0.50 respectively). On the other hand, folding, sorting and degradation; glycan biosynthesis and metabolism; nucleotide metabolism; transcription, and translation significantly decreased with ubiquity (*p*-value Kruskal-Wallis test < 0.01, linear regresion *p*-value <0.01, R^2^ = 0.43, 0.51, 0.49, 0.37 and 0.68 respectively).

Most classes showed different abundances in different environments (Fig. 3b). Only two functional classes, defense mechanisms and coenzyme metabolism, were stable (*p*-value Kruskal-Wallis test >0.01). The rest showed clear differences.

We checked that these differences were not caused by different genomic sizes of the genomes associated to each environment, by fitting a linear regression of the ratio of COGs and ORFs in each functional class to the genomic size of the corresponding genomes. (Additional files 5 and 6, respectively). Most regressions were not significant (*p* < 0.01) and therefore indicate that there is no relation between functional class abundance and genomic size in particular environments.

Regarding particular environments, host-associated habitats, such as oral and gut, were enriched in carbohydrate transport and metabolism. They also had high proportion of genes belonging to nucleotide metabolism, replication, transcription and translation, but this is probably linked to the smaller sizes of their genomes (Additional files 5 and 6). The same tendencies were observed using the KEGG database but adding a high proportion of genes devoted to membrane transport (Additional file 4). Microorganisms living in thermal environments have a high amount of genes devoted to energy metabolism, coenzyme metabolism, and posttranslational modification and chaperones. Concordantly, the same tendencies were observed in the KEGG database but adding a high proportion devoted to membrane transport in host-associated environments (Additional file 4). using KEGG database we also observe a high proportion of genes dedicated to vitamins and cofactors metabolism (Additional file 4B).

Soil microorganisms were enriched in carbohydrate metabolism, secondary metabolism, and were low in genes of the cell wall machinery and translation. In the COG database, they were also enriched in signal transduction systems (Fig. 3b).

Marine and freshwater organisms showed very similar profiles: they were enriched in motility, and secretion and signal transduction systems, while were depleted in carbohydrate metabolism (for freshwaters, this correlates with genomic size).

In concordance with previous studies, a larger percentage of cell motility genes was found in microorganisms living in open environments (thermal, marine [32], freshwaters [33] and soils [34–36]) than in host-associated microorganisms (oral and gut).

When, instead of focusing on the ratio of COGs, we considered the ratio of ORFs (that is, the total number of genes for each class, Fig. 3c-d), the trends previously found not only hold, but are augmented.

Finally, we analyzed the relationship between the presence of some genes and the ubiquity of the corresponding genomes, to determine whether some genes promoted adaptation to the different and variable conditions corresponding with diverse environments. To that end, we studied the distribution of the presence of each COG in organisms living in a given number of environments (pooling together the genera associated to that particular number of environments, and then calculating the average of the presence of each COG in the genomes of these genera). We used a linear regression between the COG presence ratio and the number of associated environments to study the presence of potential relationships between these two factors. We found that few, but some COGs were positively correlated (*p*-value <0.01, R^2^ > 0.7) with the ubiquity of their corresponding taxa. (Table 1, Additional file 7).

**Table 1:** Genes whose abundance in genomes is linearly correlated to the ubiquity of these genomes

Group	Functional class	Gene	COG	Adjusted R-squared
CELLULAR PROCESSES AND SIGNALING	Cell wall / membrane / envelope biogenesis	Sortase and related acyltransferases	COG1247	0.7484
	Posttranslational modification, protein turnover, chaperones	Glutatione peroxidase	COG0386	0.8488
	Signal transduction mechanisms	Osmosensitive K+ channel histidine kinase	COG2205	0.7662
INFORMATION STORAGE AND PROCESSING	Replication, recombination and repair	Nucleotidyltransferase/DNA polymerase involved in DNA repair	COG0389	0.73
		A/G-specific DNA glycosylase	COG1194	0.7548
	Translation, ribosomal structure and biogenesis	Acetyltransferases, including N-acetylases of ribosomal proteins	COG1670	0.7714
METABOLISM	Carbohydrate transport and metabolism	2,4-dihydroxyhept-2-ene-1,7-dioic acid aldolase	COG3836	0.7147
	Coenzyme transport and metabolism	Dihydrofolate reductase	COG0262	0.8237
	Energy production and conversion	Glycerol-3-phosphate dehydrogenase	COG0578	0.7355
		Coenzyme F420-dependent N5, N10-methylene tetrahydromethanopterin reductase and related flavin-dependent oxidoreductase	COG2141	0.7239
	Inorganic ion transport and metabolism	Phosphate/sulphate permeases	COG0306	0.7579
	Lipid transport and metabolism	Acyl CoA: acetate/3-ketoacid CoA transferase, alpha subunit	COG1788	0.7461
	Nucleotide transport and metabolism	Thymidilate synthase	COG0207	0.7334

## Discussion

### Relationships of 16S copy number, number of different 16S genes, genome size and functional profile with ubiquity and environmental preferences

The existence of a positive correlation between genomic size and ubiquity (Fig. 1), suggests that having a larger genome allow the microorganisms to be adapted to more environmental conditions, in concordance with previous studies [33, 37].

Also, since copy number of 16S rRNA gene is likely related to fast growth potential [23, 38–45], the results indicated that this potential is uncorrelated with the adaptation to different environments. Similarly, although it has been suggested that microorganisms with multiple copies of the 16S rRNA gene can be more adaptable to changing environmental conditions and grow more readily on culture media [23, 46], we found that the presence of different 16S rRNA genes did not correlate with ubiquity.

Alternative combinations of genome size and 16S rRNA copy number suggested diverse ecological strategies (growth rate and versatility, related to high copy number of 16S rRNA gene and large genome size, respectively [23]) for inhabiting different environments (Fig. 2).

The small genome size showed by host-associated (oral and gut) microorganisms is probably related to the stability of the environment, where fewer genes are required for adaptation. The extreme case is the genomic reduction experienced by symbionts and parasites [45, 47]. The high availability of nutrients may translate into an increased capacity for rapid growth [23], providing an explanation to their high 16S rRNA copy number. Nevertheless, this copy number showed a high variability, suggesting that different growth strategies may be present in this environment.

Large genomes containing many genes, and consequently having increased metabolic potential, are probably more ecologically successful in environments where resources are scarce but diverse, and where there is little penalty for slow growth [48]. The probable low growth rate suggested by the low 16S rRNA copy number in soil taxa (Fig. 1e) is supported by the abundance and dominance of slow growing oligotrophic α -Proteobacteria, such as non-symbiotic member of the Rhizobiaceae and Bradyrhizobiaceae [23, 48–50] families. Generation times in soil are thought to be longer than other environments [40]. The presence of microenvironments and, therefore, the different growth strategies in soil environment, is reflected in the high dispersion observed in their 16S rRNA copy number (Additional file 3B). That is why the differences in 16S copy number between soils and the rest of the environments are not significant. The same explanation can be applied to freshwaters, that showed a similar trend, although with smaller genome sizes than in soils but not significantly different from the rest of the environments (Additional file 3C), suggesting a wide range of variation.

Genome size in thermophilic microorganisms may be an indirect target of selection due to its association with cell volume. Known changes in cell structure and physiology at high temperature can provide a selective advantage to reduce cell volume [51]. Also, the apparent stability of these environments can promote smaller genome sizes, as mentioned above. The low 16S copy number can be explained by the stress conditions that penalize growth. In this regard, it is known that generation time increases when increasing habitat temperature [51].

Within the wide diversity of ecological strategies in seawater, oligotrophic bacteria are abundant [51]. These microbes grow slowly, in opposition to the fast-growing copiotrophs [52]. Despite their abundance, probably just a few taxa are oligotrophs, with small genomes and slow growth [53]. But most of the low-abundance taxa in the ocean could be capable of slow growth in energy-limited environments, and rapid growth in energy-rich environments, as suggested by their high copy number of 16S rRNA [52]. Accordingly, high growth rates occur periodically in nutrient-rich microzones [53–55]. Thus, the slow average-growth rates that are typically observed for pelagic communities do not preclude the possibility that a small fraction of the assemblage might be growing rapidly [56–58]. Copy numbers of the 16S rRNA gene show a high variability, which can explain the diversity of ecological strategies due to the presence of pelagic microniches in seemingly homogeneous ocean waters [33, 59]. Thus, many genera showed both a high copy number of 16S rRNA and a large genome, which may explain their capability of adapting to different situations. Both freshwaters and marine microorganisms presented a similar tendency in relation to 16S rRNA gene copy number and genome size (wide range of values), reflected in the non-significant differences between them and the rest of microorganisms (Additional file 3C and E).

Hence, we have found different combinations of genome size and 16S rRNA copy number that evidence different ecological strategies for inhabiting different environments (Fig. 2), supporting previous studies (soils, freshwaters and thermal environments [48, 51]) or finding novel patterns for marine environments [51].

Finally, it has been proposed that a higher number of different 16S rRNA genes makes microorganisms more adaptable to variable conditions [23, 46], and therefore environments which display more stable conditions will harbor bacteria with fewer rRNA operons [60]. However, our results do not support this hypothesis (Fig. 1f): microorganisms related to environments with a high availability of nutrients (gut and oral) presented a high number of different 16S rRNA genes, and also marine microorganisms.

### Relationships among gene/functional class content, ubiquity and environmental preferences

In ubiquitous taxa, we observed an increase in the share of functional classes proposed to be more linked to environmental adaptation, such as inorganic ion transport and metabolism, secondary metabolism, xenobiotics biodegradation and metabolism and, to a lesser extent, signal transduction [21]. In contrast, the decrease of functions related to nucleotide metabolism, replication and translation in these ubiquitous genomes is significant and may be explained by the strong positive correlation between ubiquity and genome size. As the machinery related to nucleotide metabolism, replication and translation is well conserved for all organisms, the amount of genes for these functional classes is similar for all genomes, but the ratio is smaller in these with larger genomes.

The enrichment in carbohydrate transport and metabolism observed in host-associated genomes, such as oral and gut, is in agreement with the large amount of carbohydrates present in such environments [60, 61]. Organisms living in these environments also have high ratio of genes belonging to nucleotide metabolism, replication and translation because they tend to have small genomes. We observed the same trend for organisms living in thermal environments. These also have a high amount of genes devoted to energy metabolism, and to posttranslational modification and chaperones, probably to increase protein survival and maintenance. In this habitat, carbohydrate metabolism is minimal, probably a consequence of the autotrophic metabolism found in these environments.

The enrichment in carbohydrate metabolism in soil microorganisms may be explained by the different microenvironments found in soils, with many diverse nutrients and conditions [23, 62]. They are also enriched in secondary metabolism, which is probably related to the diverse set of potential nutrients available in the soils. Interestingly, they are short in genes of the cell wall machinery and translation. Both functional classes have been demonstrated to be functional indicators of copiotrophy [63], and their depletion is in concordance with the abundance and dominance of slow-growing oligotrophic α-Proteobacteria.

Marine and freshwater organisms showed very similar profiles, as we could also see when studying 16S rRNA gene copy number and genome size, and it is tempting to hypothesize that they face similar environmental challenges. They are enriched in motility, and secretion and signal transduction systems, while depleted in carbohydrate metabolism, perhaps due to the abundance of autotrophic taxa in the photic zone, such as the phototroph *Prochlorococcus.* Also microorganisms in marine or freshwater sediments contribute to the anaerobic autotrophic metabolism.

When, instead of focusing on the number of different COGs/KEGGs, we studied the total number of ORFs belonging to these COGs/KEGGs (Fig. 3c-d and Additional file 4C-D), some interesting features that were obscured in the previous analysis emerged. For instance, while lipid metabolism was very stable when considering number of different COGs, there was a large increase in the number of genes related to this function in marine, freshwaters, and especially soil environments. That is, gene families in these classes have undergone expansion by duplication resulting in paralogs. Also there was a large increase in the transcription class in soils, because of the expansion of the COGs related to transcription factors. This is related to the creation of new regulatory proteins conferring increased fine-tuning of gene expression [64].

The negative linear correlation found between translation, ribosomal structure and biogenesis and genome size (*p*-value <0.01, R^2^ > 0.7) for all environments is probably due to that the machinery needed for this process is highly conserved in all microorganisms, and therefore the ratio is smaller in bigger genomes.

Finally, ubiquity, and consequently adaptation, are promoted by strategies of pioneering, colonization, and protection from diverse stresses. This is noticeable by the positive relationship between ubiquity and the presence of genes like sortases and related acyltransferases, involved in cell wall/membrane/envelope biogenesis, strategies for bacterial escape from the host’s immune response and creation of biofilms [65], that can promote colonization processes. Many genes related to protection from diverse stresses were also found: signal transduction proteins such as the osmosensitive k + channel histidine kinase, which has an important role in osmoregulation and the chemotaxis system, participating in control complex processes such as the initiation of development in microorganisms; histidine kinase two-component systems, that are extremely common in bacteria and play an important role in signal transduction that is essential for adaptation to bacterial stress [66]; gluthatione peroxidase, whose main biological role is to protect the organism from oxidative damage, related to posttranslational modification protein turnover chaperones [67]. And finally, nucleotidyltransferase/DNA polymerase and A/G-specific DNA glycosylases, both involved in DNA repair [68, 69].

## Conclusions

The study of the relationships between 16S copy number, genome size and adaptation to diverse environments showed that adaptation was linked to genome size [37, 38]. In contrast, 16S copy number was not directly related to ubiquity, highlighting that growth potential (related to 16S copy number [23, 40–46]) does not favor the ubiquity. We observed distinctive trends regarding these two features: Small genome size and low 16S copy number, found in thermal environments. This was probably the result of a direct targeting of natural selection on cell size, because their metabolic analysis suggested that large cells could suffer significant fitness costs at high temperatures [51]. Low genome size and high 16S copy number was found in host-associated habitats, as a result of the stability of the environment and the potential high growth rate of the organisms in them.

Larger genome sizes were found in environmental organisms from soils, freshwaters and the sea. The first two had a low 16S copy number. This is in accordance with complex environments with many diverse niches, where organisms tend to be slow growers. Large genomes are supposed to be more ecologically successful in environments where resources are scarce but diverse and where there is little penalty for slow growth [48].

Finally, large genomes with high 16S copy number were found in marine environments. This is probably related to the capacity of marine microorganisms for rapid growth in appropriate conditions. We hypothesize that, although most of marine microorganisms are oligotrophs, they consist on only a few taxa. In contrast, the majority of the taxa in the sea may be, in fact, copiotrophs, increasing their growth when a higher quantity of nutrients is available.

However, although marine microorganisms tend to have higher copy number of 16S rRNA gene than soils and freshwaters, these differences were not significant (Additional file 3), as well as the differences in genome size of freshwater and marine microorganisms, suggesting a wide range of variation in both genome size and 16S copy number explained by the presence of microniches in these three environments.

The analysis of functional classes showed some clear signatures in different environments, as described previously [21]. For example, a higher presence of carbohydrate metabolism and defense mechanisms in gut and oral cavity, reflecting the availability of nutrients and the need of defense mechanisms against the host immune system, and the fact that mobility may not be necessary in these environments. Another example is the high fraction of COGs devoted to nucleic acid functions in thermophilic microorganisms, which could be due to the need of frequent repair of DNA damage caused by the extreme conditions. Regarding the number of environments in which the organisms were able to live in, there were almost no differences between functional classes. Only few classes were correlated with the adaptation to a high number of environments: inorganic ion transport and metabolism, secondary metabolism, and signal transduction.

## Additional files

Additional file 1: Procedure for obtaining associations between taxa and environments. Only the genera significantly related to some environment are shown in the Fisher’s exact test table. ID. (PDF 18 kb)

Additional file 2: Number of genomes associated to each environment and to each number of environments. (XLSX 5 kb)

Additional file 3: Plots relating the average genome size of every genus to their average number of 16S rRNA genes. All genera are shown, highlighting these associated to particular environments: (A) Host-associated (B) Soil samples. (C) Freshwater samples. (D) Thermal samples (E) Marine samples (.pdf format). The *p*-values of the Mann-Whitney test applied to the genome size and 16S rRNA gene copy number between the genera associated to a particular environment and the rest are provided in the figures. (PDF 758 kb)

Additional file 4: Fraction of distinct KEGGs (A and B) and ORFs (C and D) dedicated to different functional classes regarding their ubiquity (A and C), and the preferred environment for each genus (B and D). The error bars represent the range of the data. (PDF 118 kb)

Additional file 5: Linear regressions between COG ratios and genome size, for genera with different environmental preferences. Only significant instances (*p*-value <0.01) are shown. (PDF 199 kb)

Additional file 6: Linear regressions between ORF ratio and genome size for genera with different environmental preferences. Only significant instances (*p*-value <0.01) are shown. (PDF 176 kb)

Additional file 7: Linear regression between the number of environments associated to the genera, and the average presence of some COGs in these genera, showing linear relationships between presence of some genes and the ubiquity of the corresponding genomes (.pdf format) (A) Sortase and related acyltransferases (B) Glutatione peroxidase. (C) Osmosensitive K+ channel histidine kinase (D) Nucleotidyltransferase/DNA polymerase involved in DNA repair (E) A/G-specific DNA glycosylase (F) Acetyltransferases, including N-acetylases of ribosomal proteins (G) 2,4-dihydroxyhept-2-ene-1,7-dioic acid aldolase (H) Dihydrofolate reductase (I) Glycerol-3-phosphate dehydrogenase (J) Coenzyme F420-dependent N5,N10-methylene tetrahydromethanopterin reductase and related flavin-dependent oxidoreductase (K) Phosphate/sulphate permeases (L) Acyl CoA: acetate/3-ketoacid CoA transferase, alpha subunit (M) Thymidilate synthase. (PDF 175 kb)

## Abbreviations

COG: Clusters of Orthologous Groups
KEGG: Kyoto enciclopedia of genes and genomes
NCBI: National center for biotechlology information
ORF: Open reading frame
