# Supplementary material for: Relating genomic characteristics to environmental preferences and ubiquity in different microbial taxa
Source: BMC Genomics. 2017 Jun 29;18:499. doi: 10.1186/s12864-017-3888-y (PMC5492924; doi:10.1186/s12864-017-3888-y)
Supplement: Supplementary file 1 — Procedure for obtaining associations between taxa and environments. Only the genera significantly related to some environment are shown in the Fisher’s exact test table. ID. (PDF 18 kb) [file 12864_2017_3888_MOESM1_ESM.pdf]

Combined table

ID sample	Genus	Environment
1394	Acidianus	Freshwaters
9372	Acidianus	Freshwaters
14670	Acidianus	Freshwaters
1368	Acidianus	Thermal
1391	Acidianus	Thermal
7073	Acidianus	Thermal
7074	Acidianus	Thermal
7075	Acidianus	Thermal
7078	Acidianus	Thermal
9179	Acidianus	Thermal

Co-occurrence matrix

Genus	Environment	Co-ocurrences
Acidianus	Freshwaters	3
Acidianus	Thermal	7

Fisher's exact test

Genus	Environment	Co-ocurrences	P-value
Acidianus	Thermal	7	0.006640674

Genus-ubiquity matrix

Genus	Ubiquity
Acidianus	2
