# Supplementary figures and images for: Relating genomic characteristics to environmental preferences and ubiquity in different microbial taxa
Source: BMC Genomics. 2017 Jun 29;18:499. doi: 10.1186/s12864-017-3888-y (PMC5492924; doi:10.1186/s12864-017-3888-y)

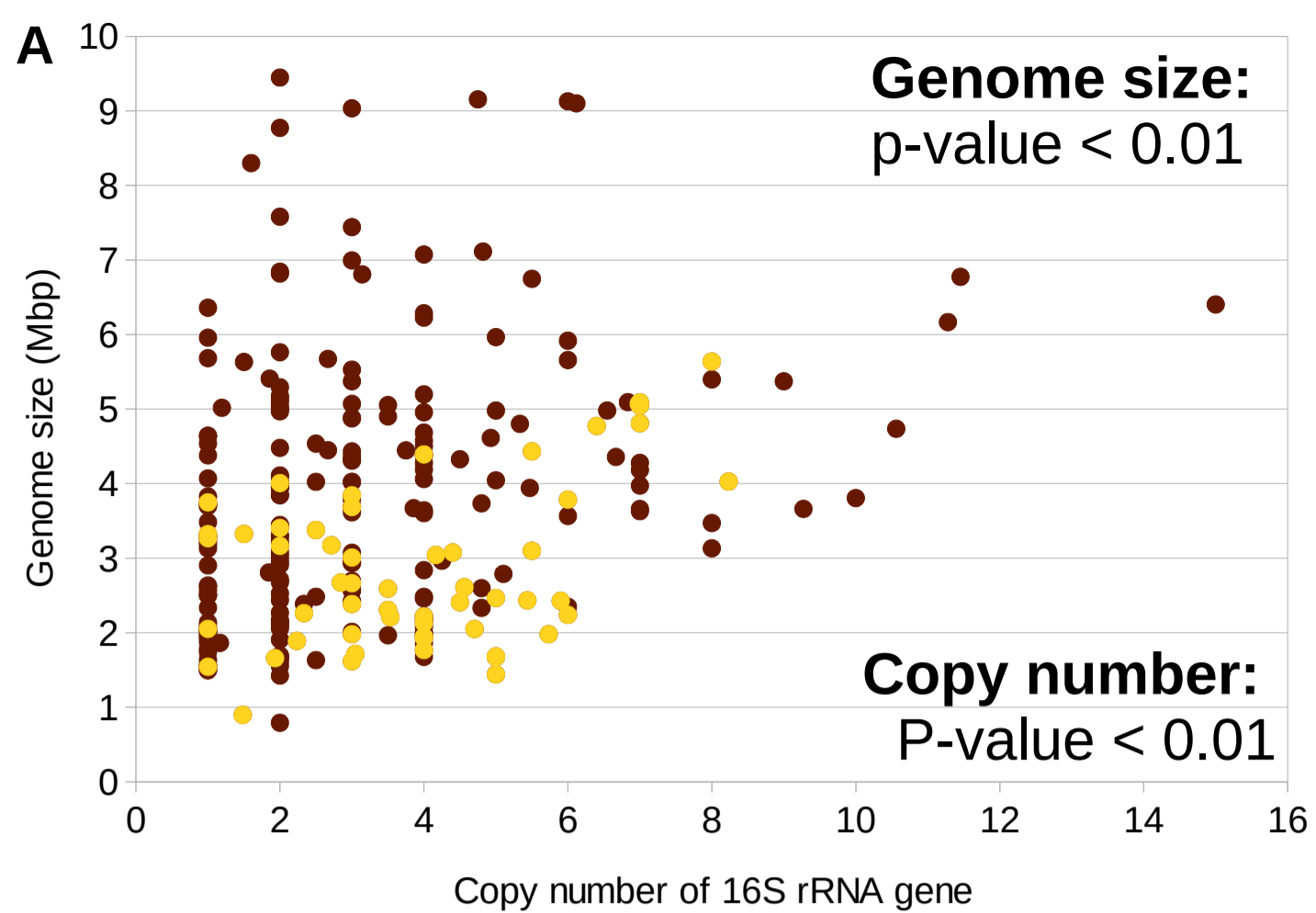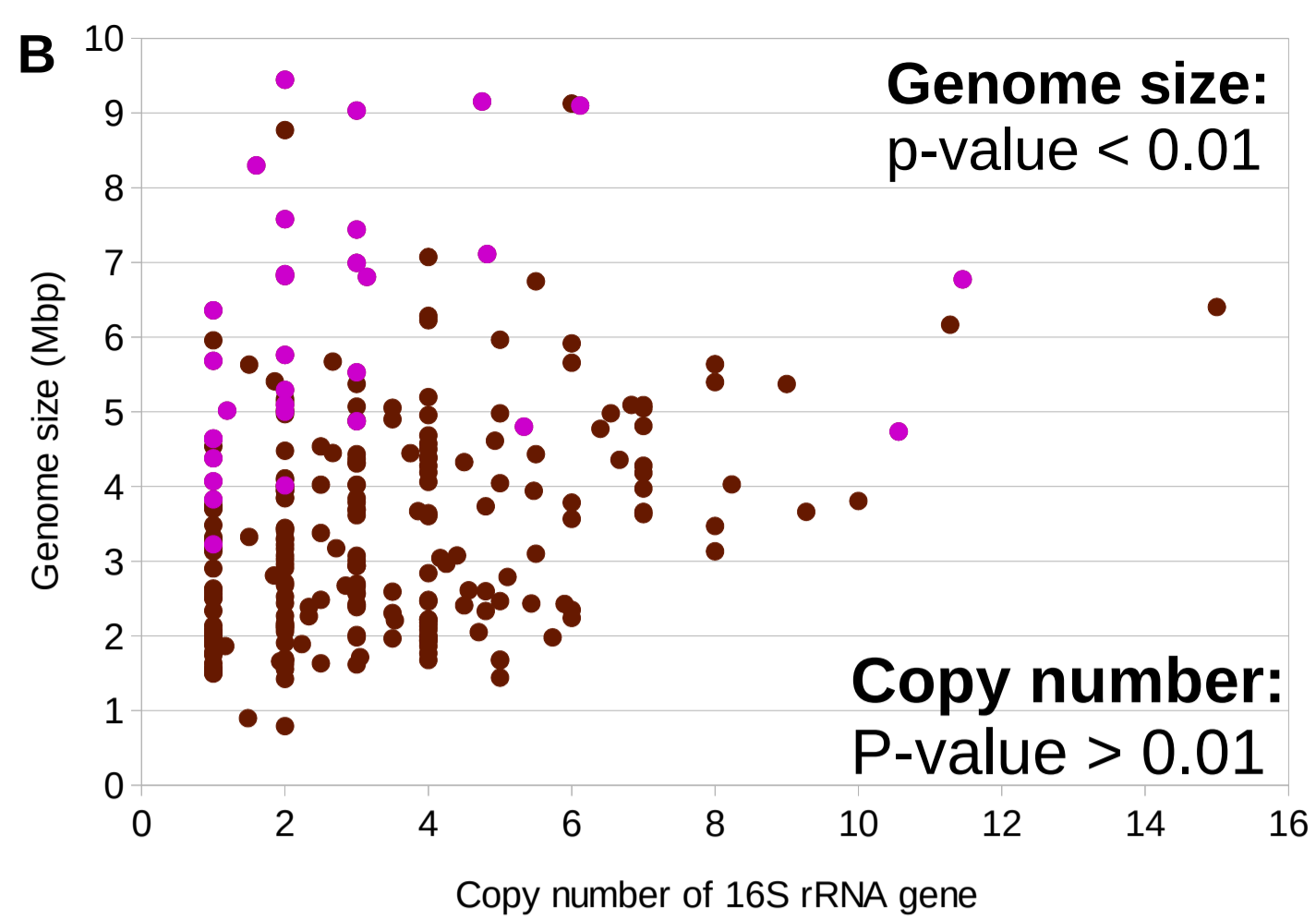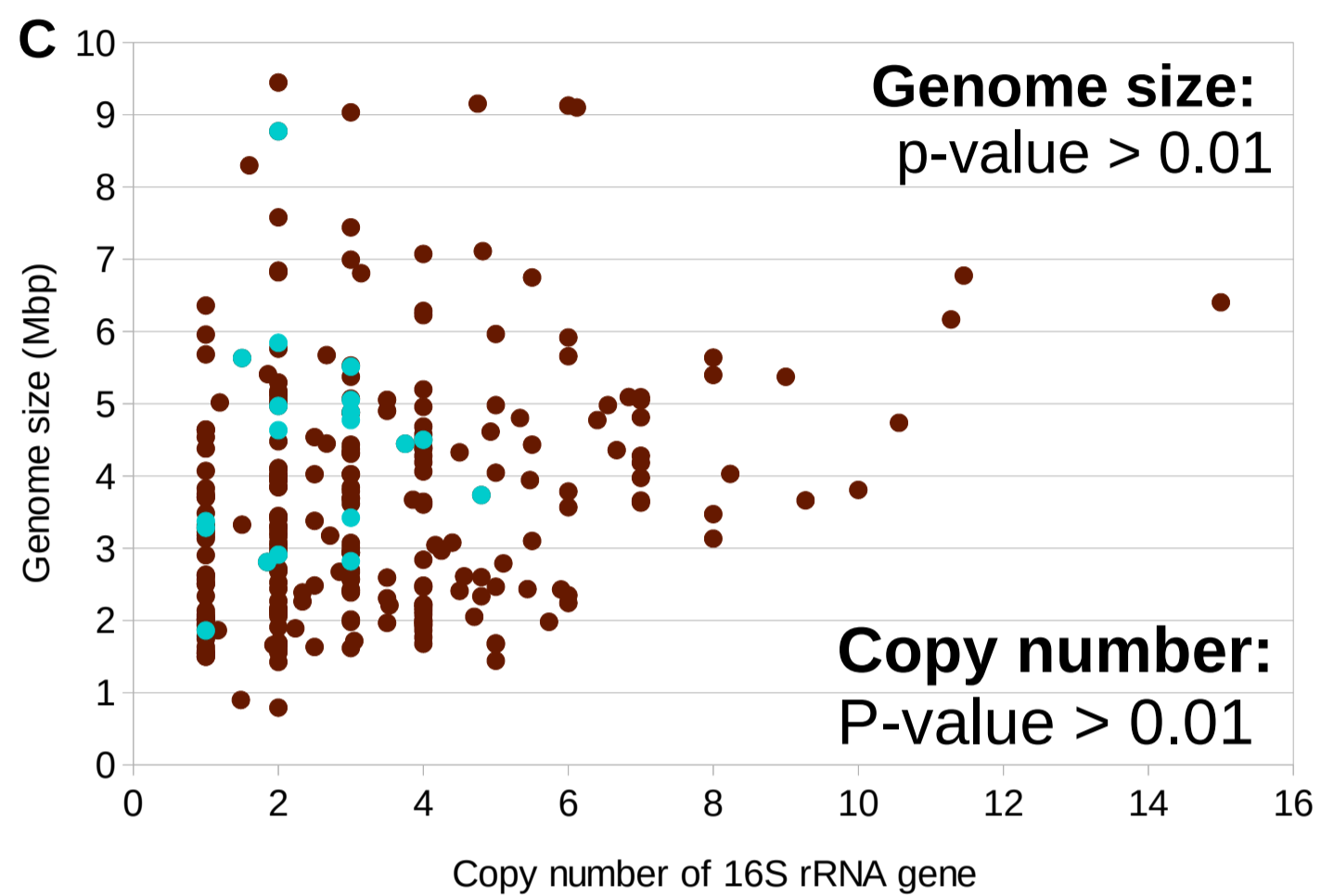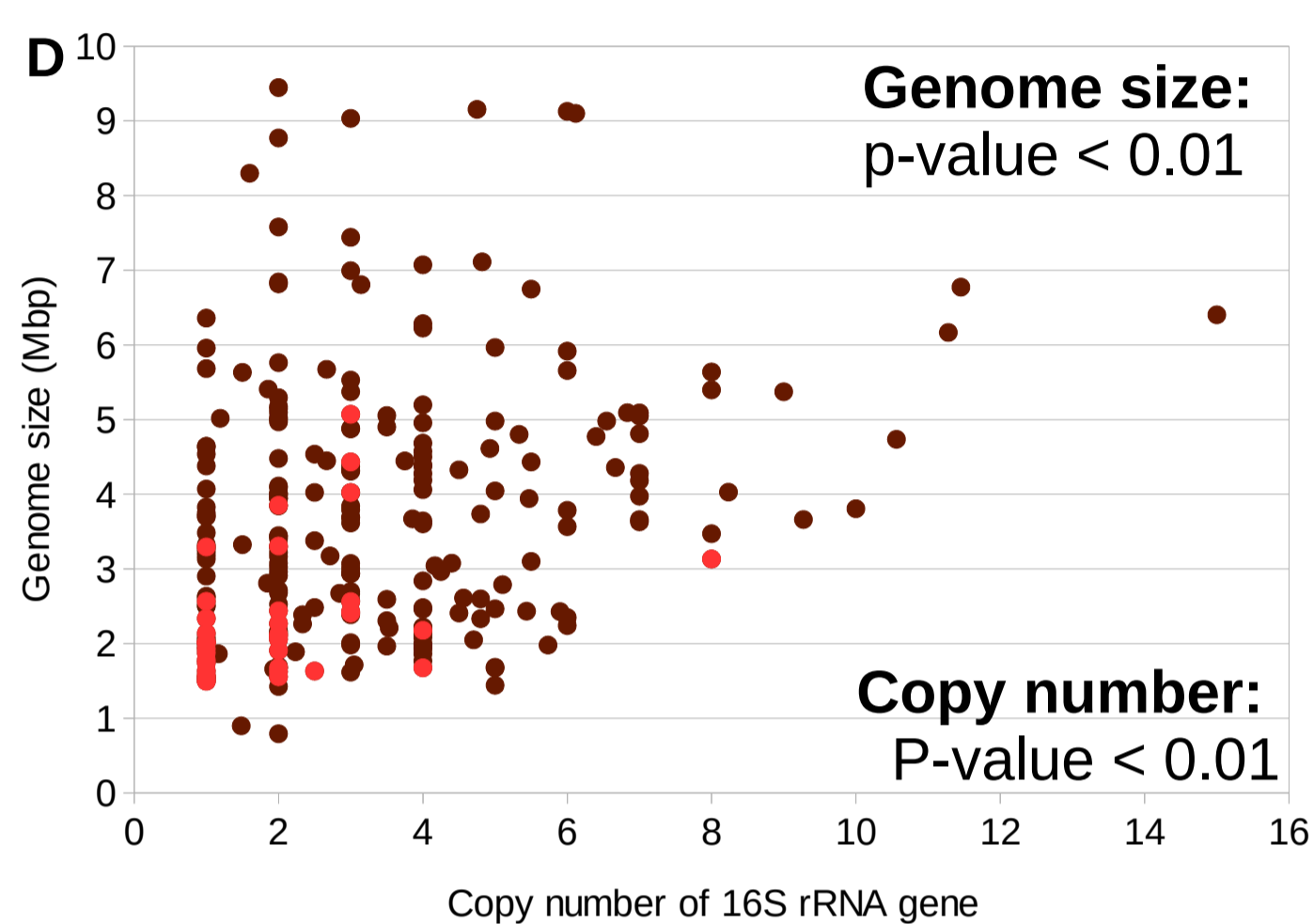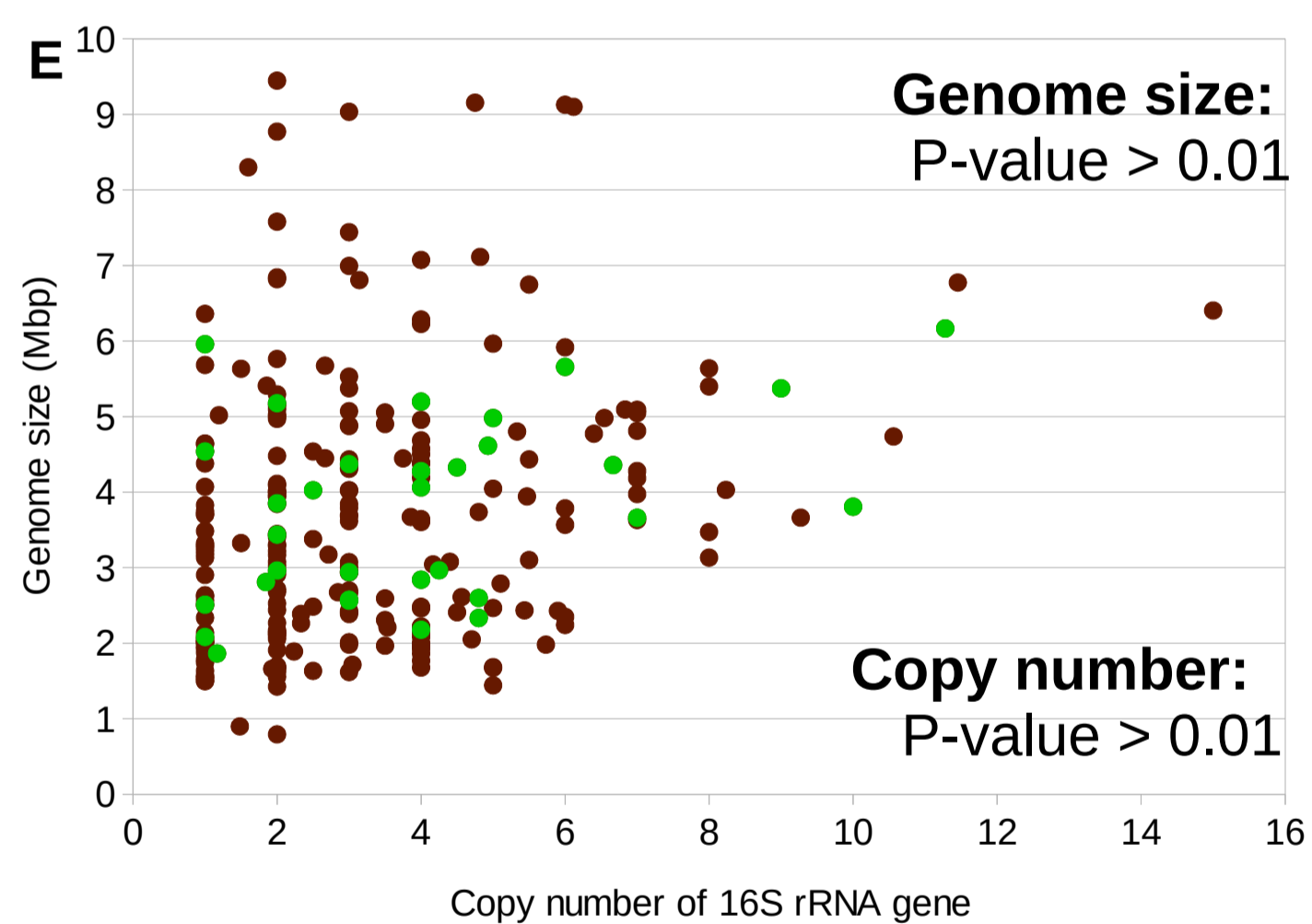

- Host-associated
- Soils
- Freshwaters
- Thermal
- Marine
- Other

Supplement: Supplementary file 3 — Plots relating the average genome size of every genus to their average number of 16S rRNA genes. All genera are shown, highlighting these associated to particular environments: (A) Host-associated (B) Soil samples. (C) Freshwater samples. (D) Thermal samples (E) Marine samples (.pdf format). The p-values of the Mann-Whitney test applied to the genome size and 16S rRNA gene copy number between the genera associated to a particular environment and the rest are provided in the figures. (PDF 758 kb) [file 12864_2017_3888_MOESM3_ESM.pdf]

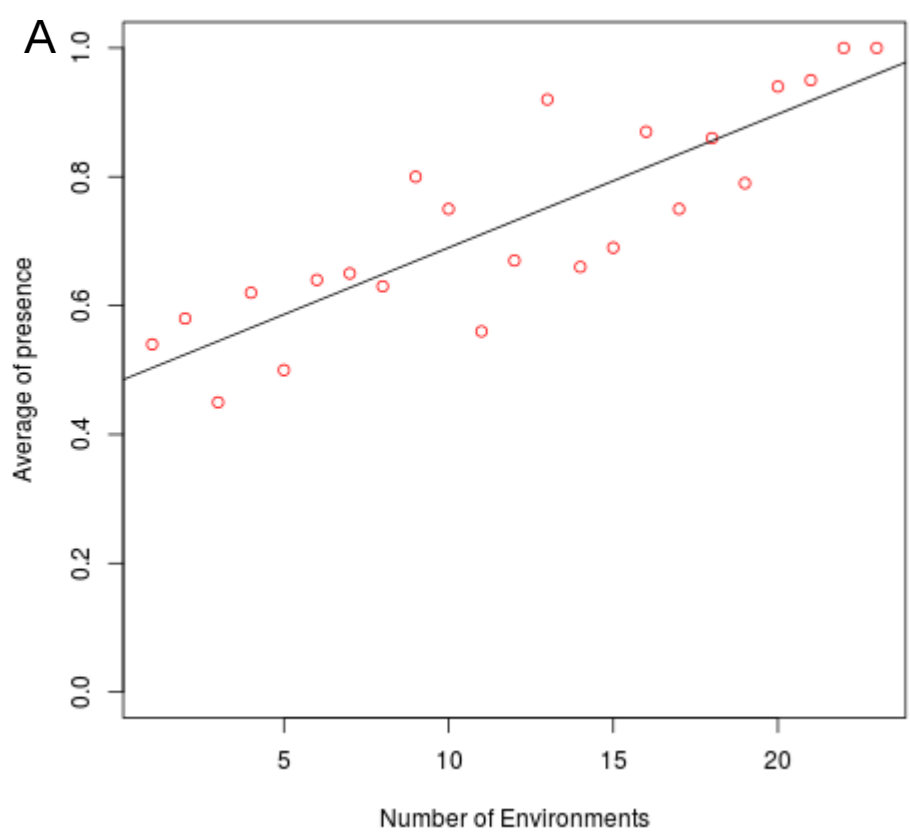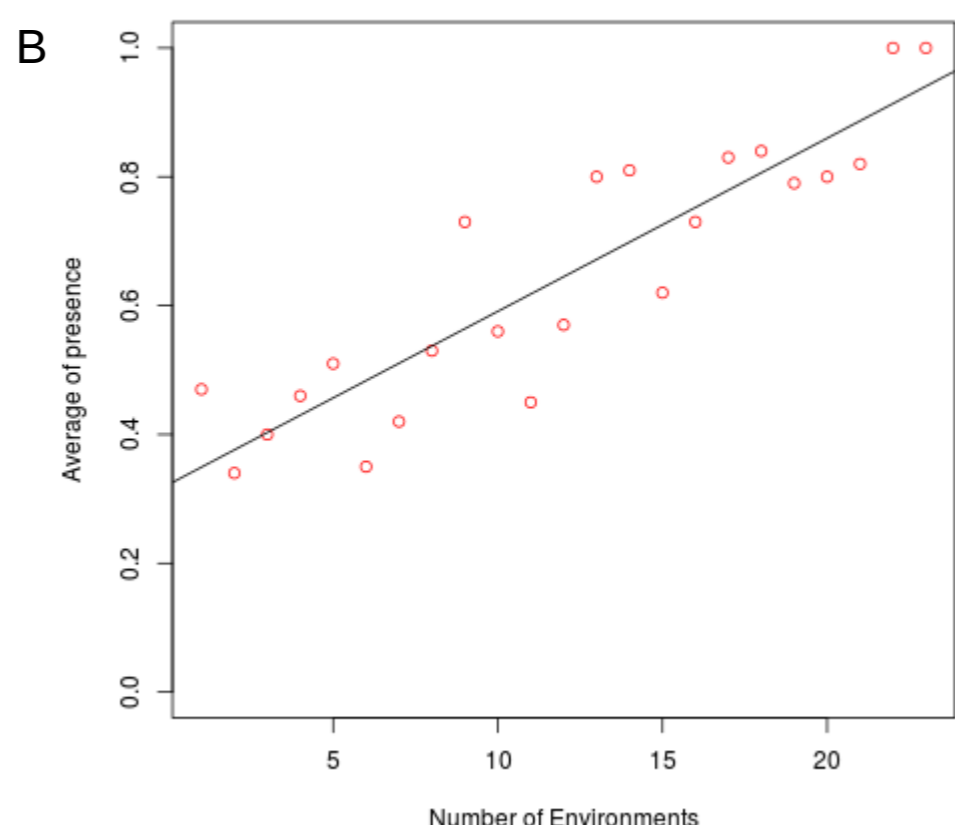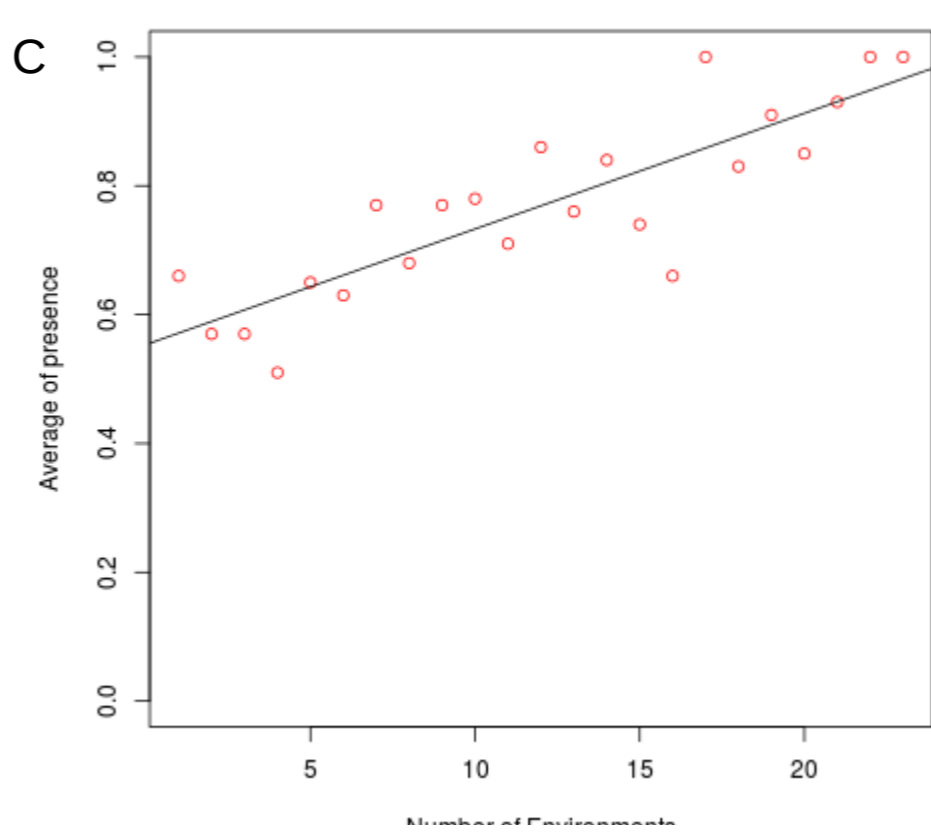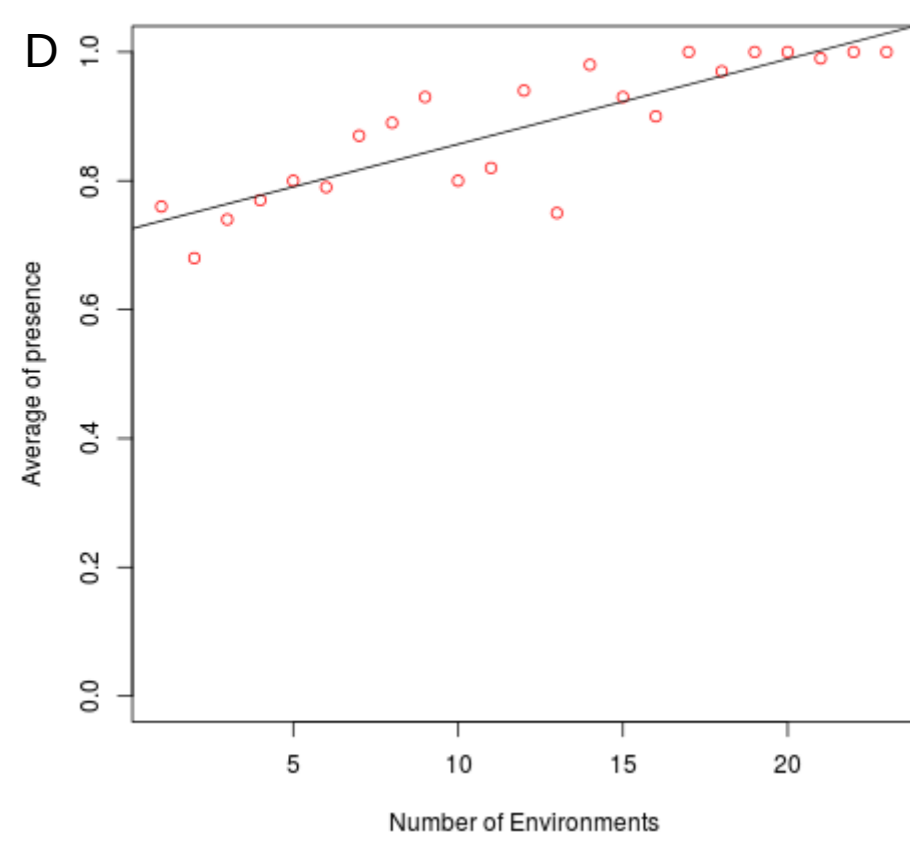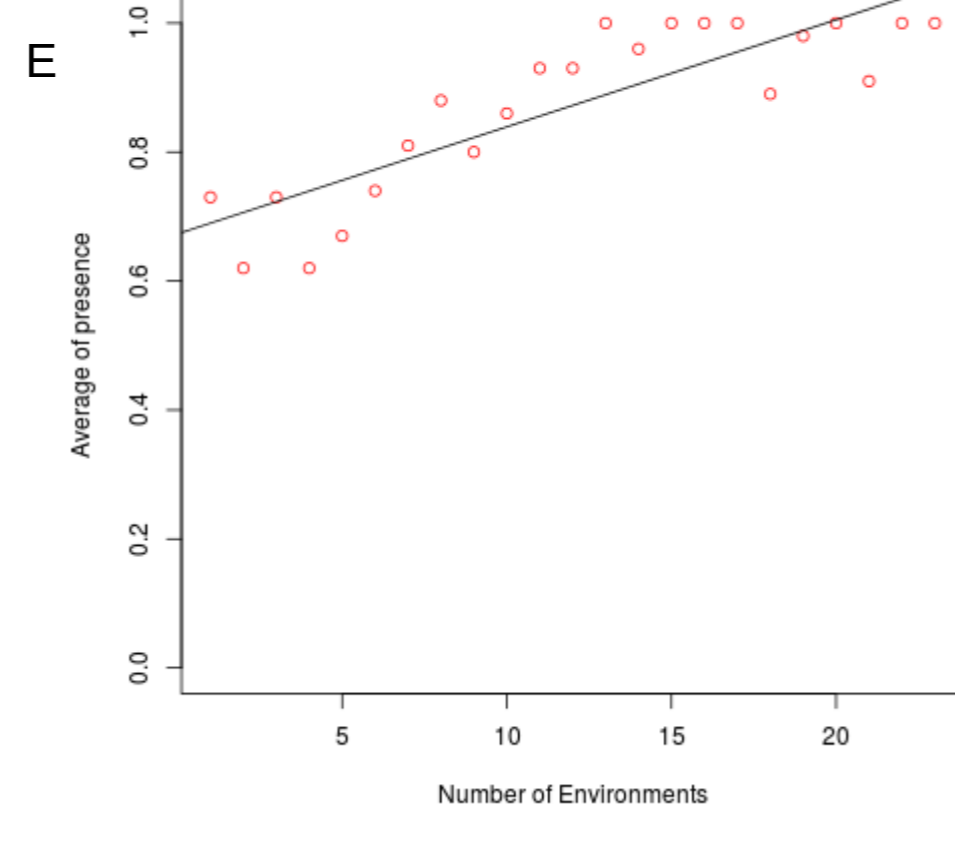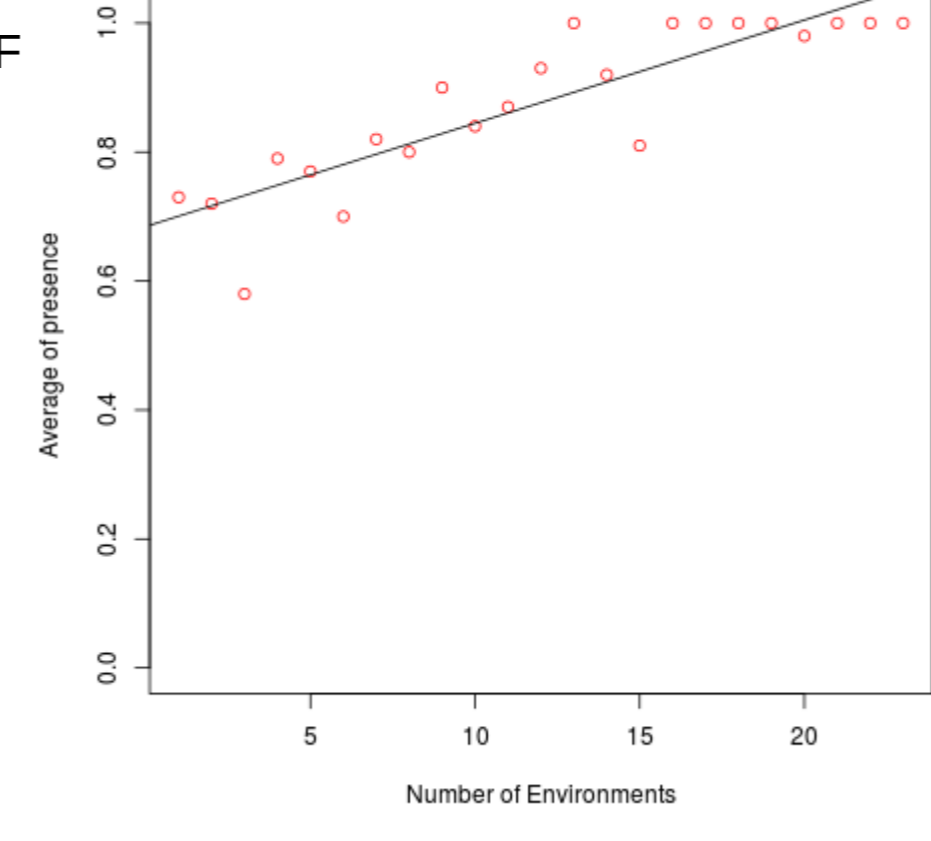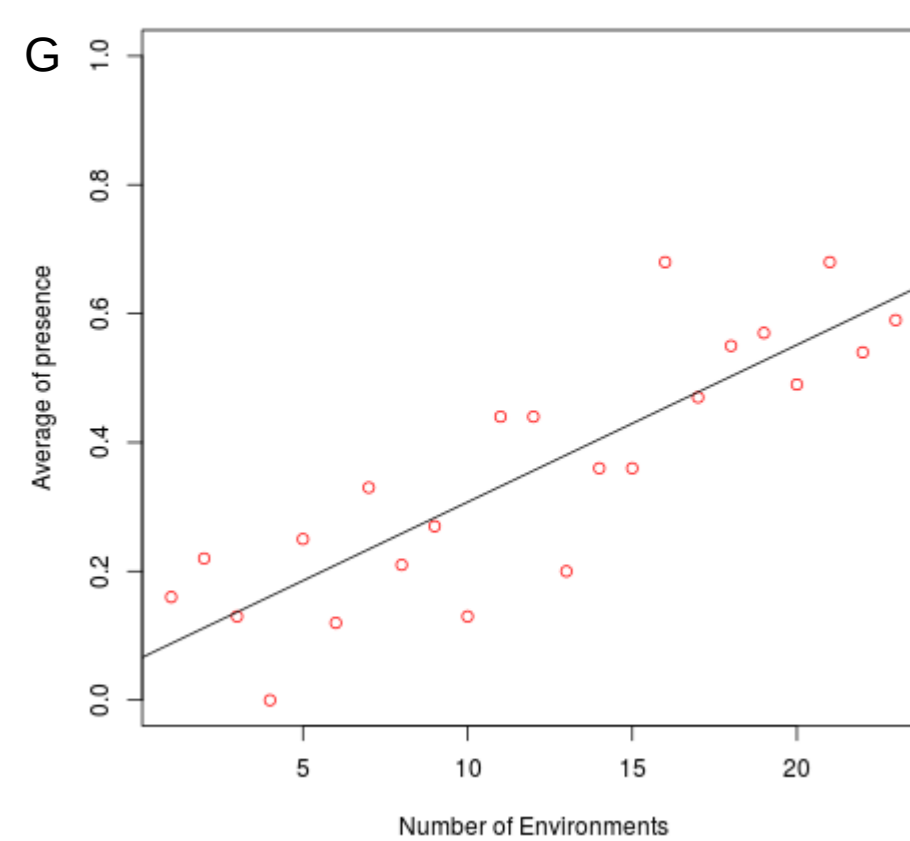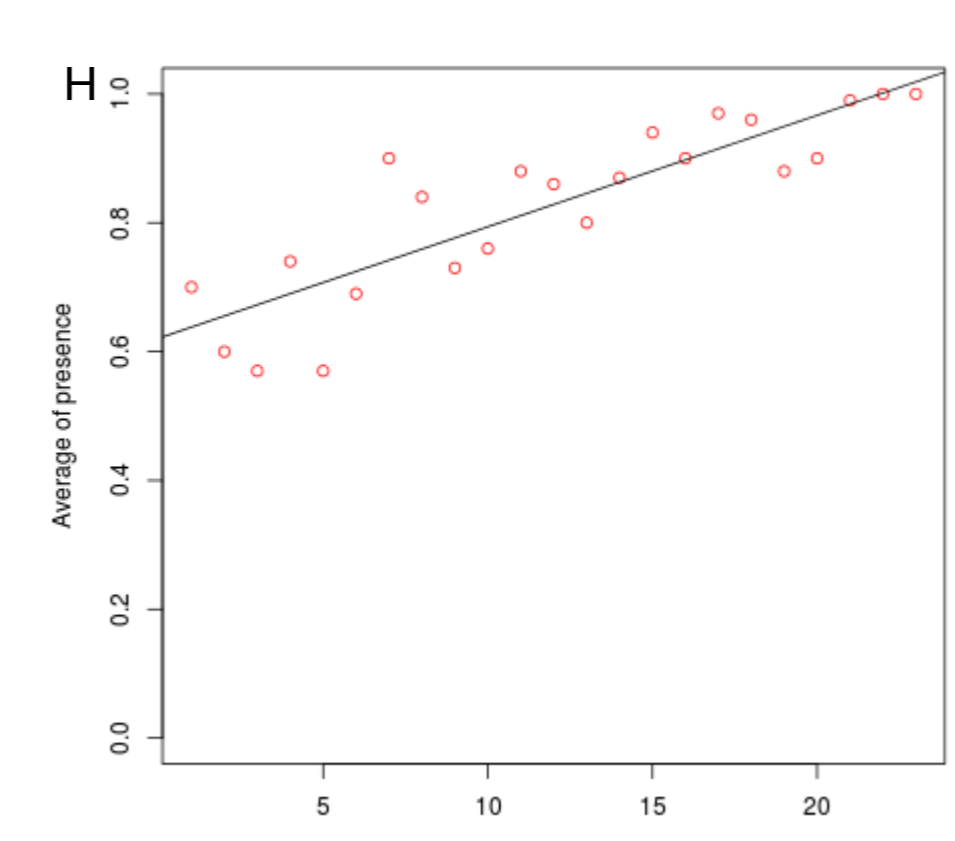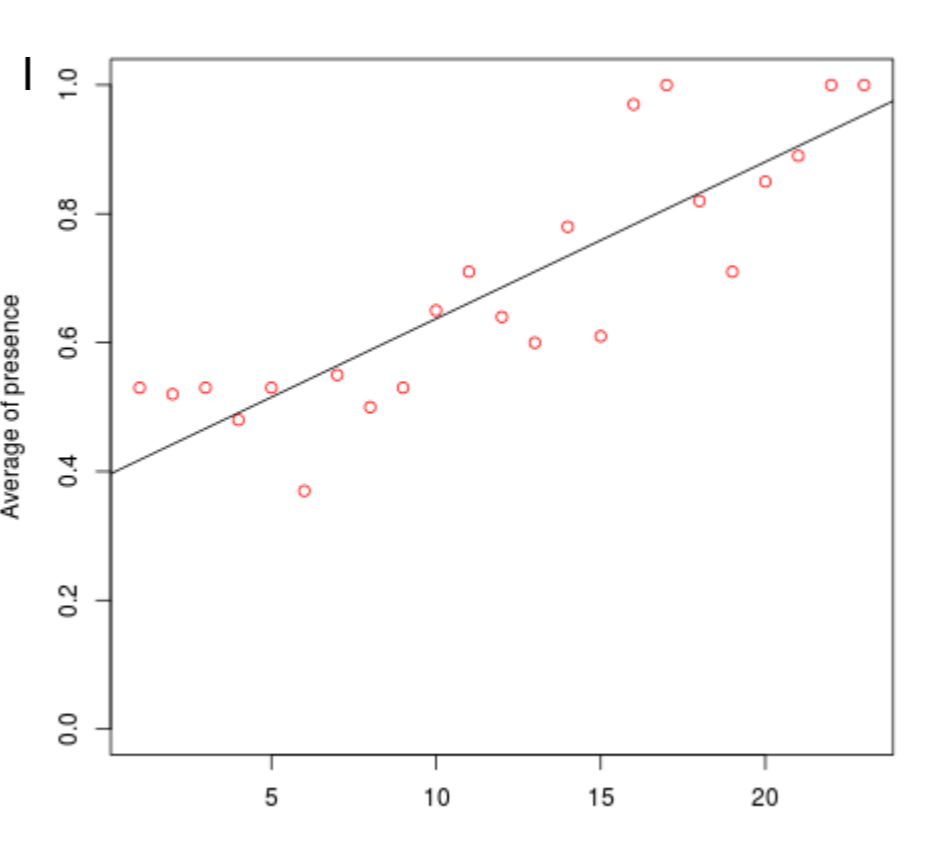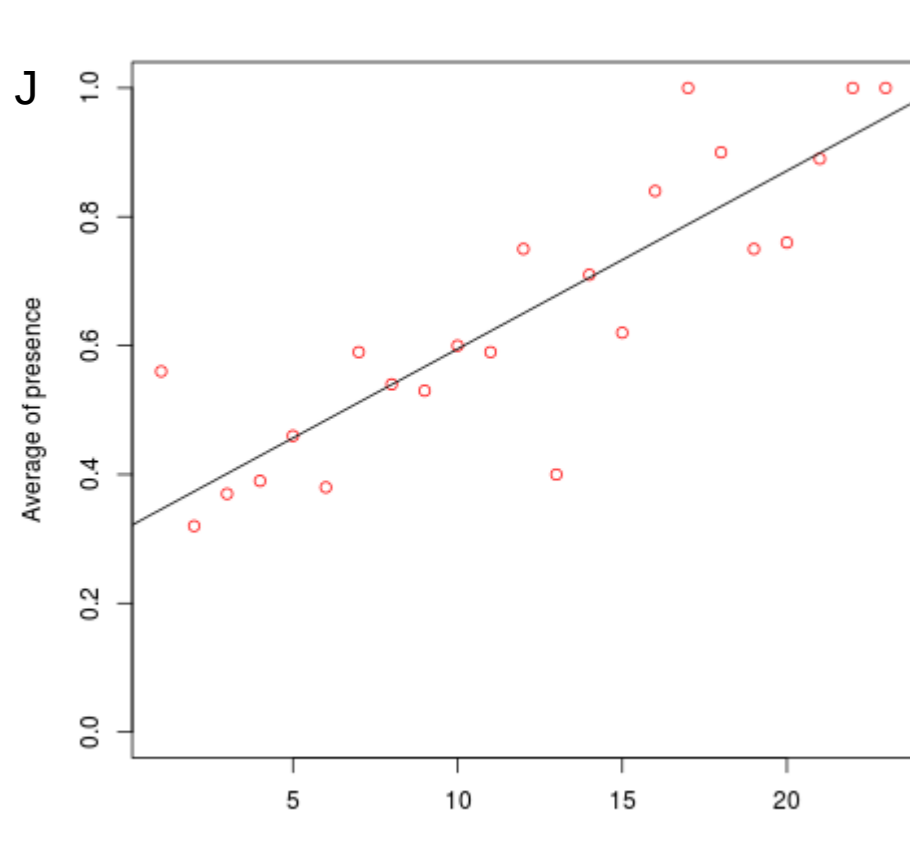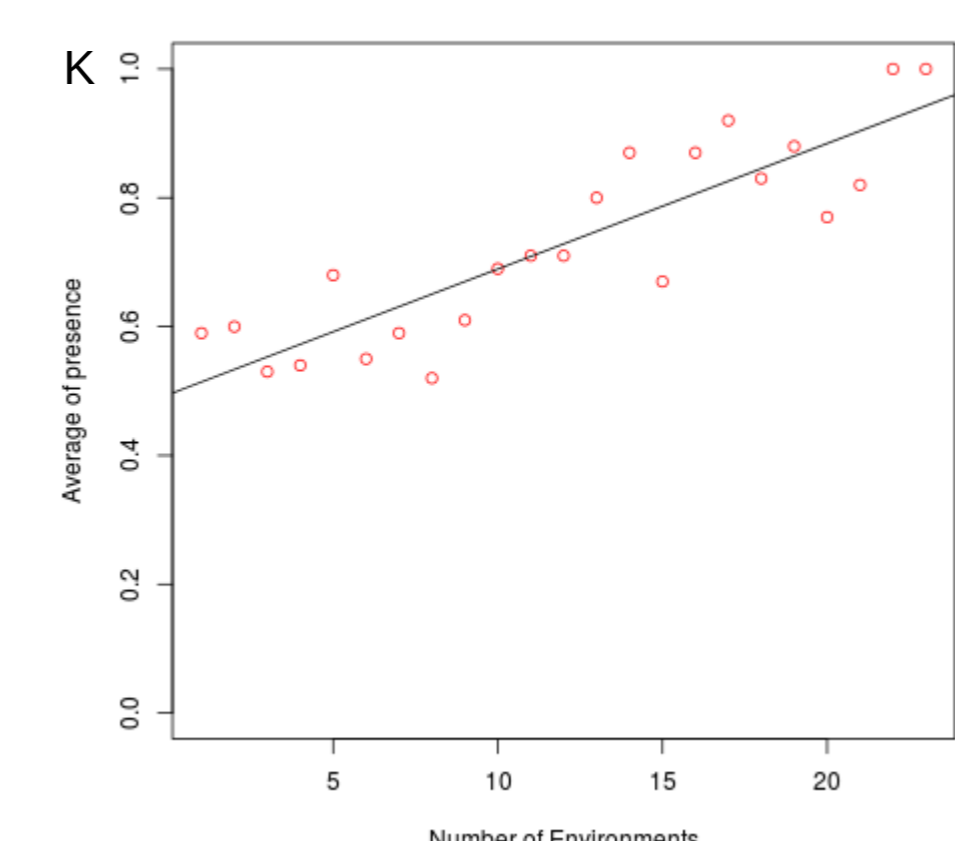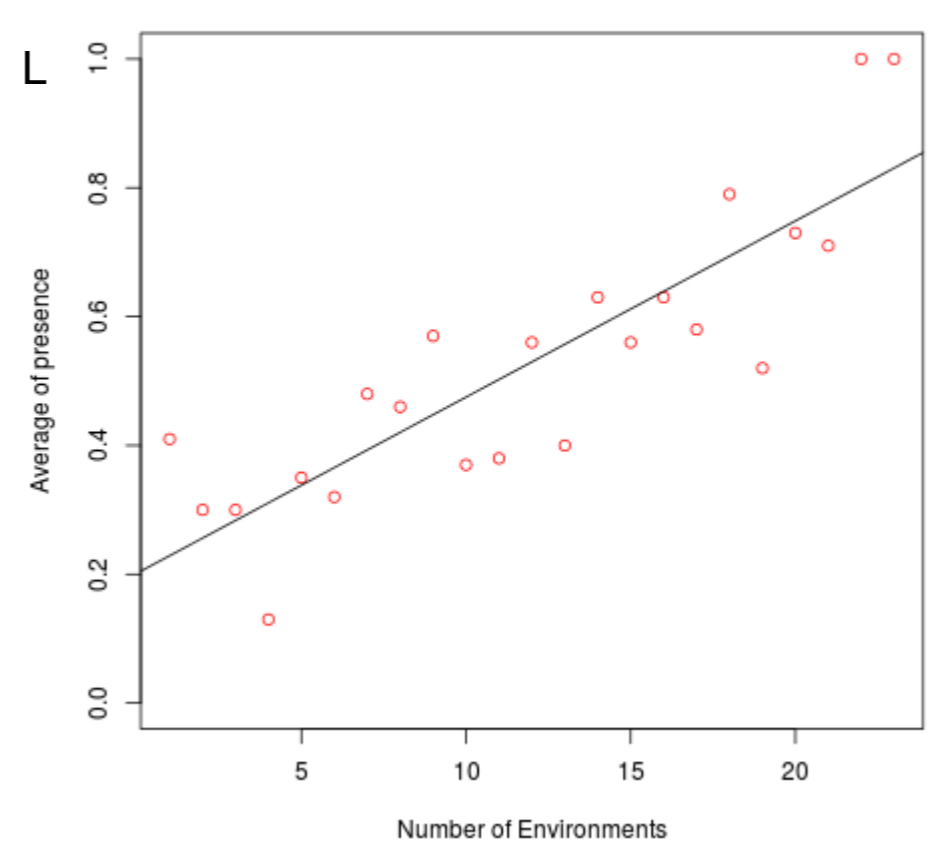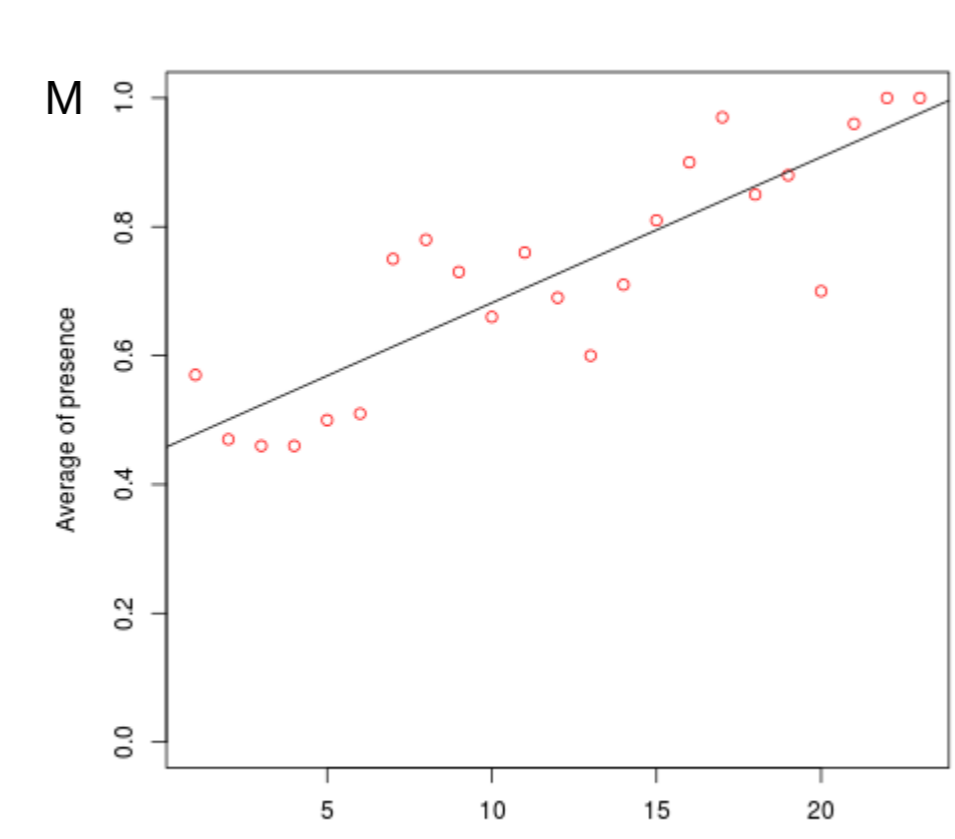

Supplement: Supplementary file 7 — Linear regression between the number of environments associated to the genera, and the average presence of some COGs in these genera, showing linear relationships between presence of some genes and the ubiquity of the corresponding genomes (.pdf format) (A) Sortase and related acyltransferases (B) Glutatione peroxidase. (C) Osmosensitive K+ channel histidine kinase (D) Nucleotidyltransferase/DNA polymerase involved in DNA repair (E) A/G-specific DNA glycosylase (F) Acetyltransferases, including N-acetylases of ribosomal proteins (G) 2,4-dihydroxyhept-2-ene-1,7-dioic acid aldolase (H) Dihydrofolate reductase (I) Glycerol-3-phosphate dehydrogenase (J) Coenzyme F420-dependent N5,N10-methylene tetrahydromethanopterin reductase and related flavin-dependent oxidoreductase (K) Phosphate/sulphate permeases (L) Acyl CoA: acetate/3-ketoacid CoA transferase, alpha subunit (M) Thymidilate synthase. (PDF 175 kb) [file 12864_2017_3888_MOESM7_ESM.pdf]
