# Supplementary material for: Relating genomic characteristics to environmental preferences and ubiquity in different microbial taxa
Source: BMC Genomics. 2017 Jun 29;18:499. doi: 10.1186/s12864-017-3888-y (PMC5492924; doi:10.1186/s12864-017-3888-y)
Supplement: Supplementary file 4 — Fraction of distinct KEGGs (A and B) and ORFs (C and D) dedicated to different functional classes regarding their ubiquity (A and C), and the preferred environment for each genus (B and D). The error bars represent the range of the data. (PDF 118 kb) [file 12864_2017_3888_MOESM4_ESM.pdf]

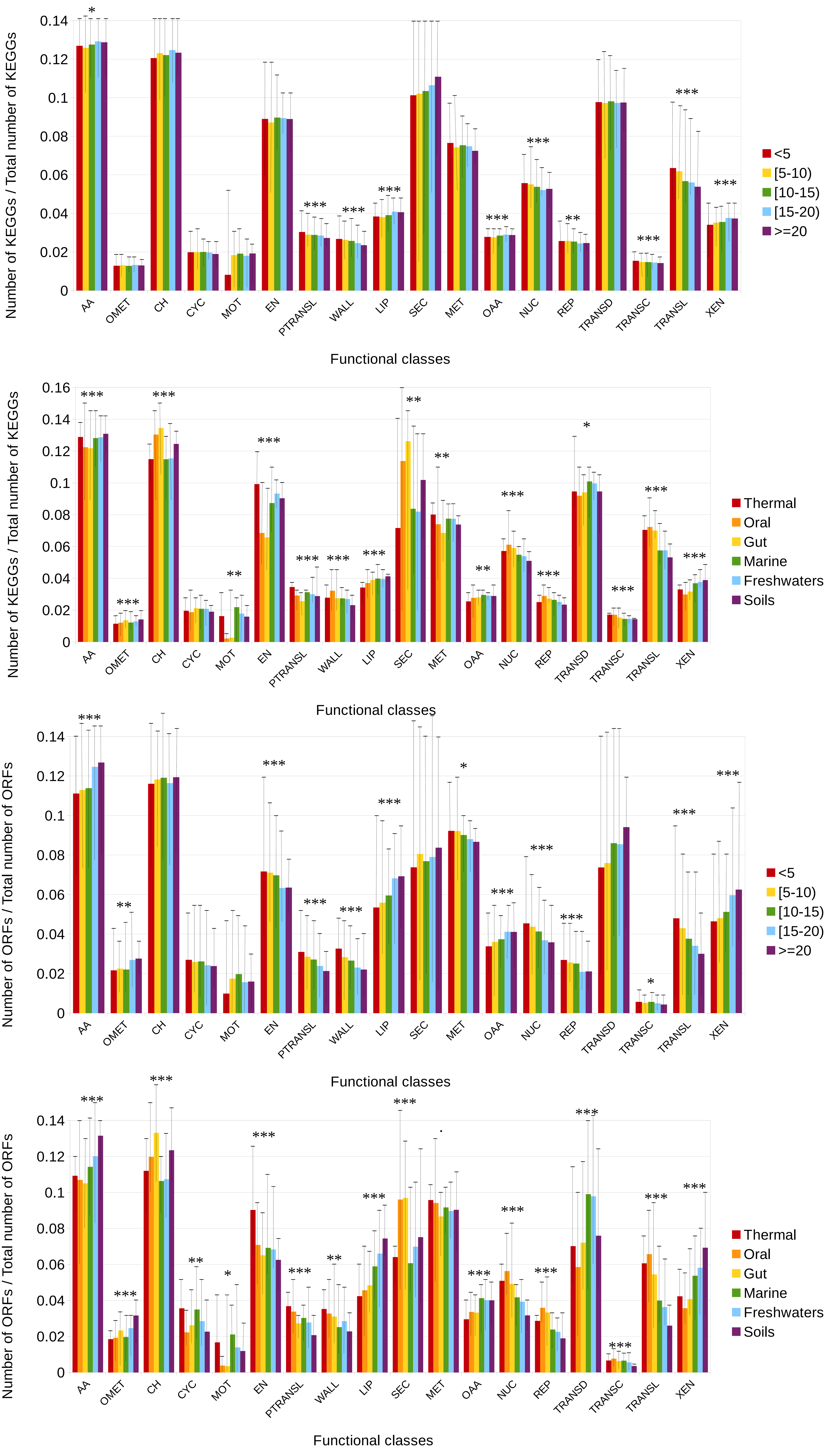

AA: Aminoacid Metabolism. OMET: Biosynthesis of other secondary metabolites. CH: Carbohydrate metabolism. CYC: Cell growth and death. MOT: Cell motility. EN: Energy metabolism. PTRANSL: Folding, sorting and degradation. WALL: Glycan biosynthesis and metabolism; LIP: Lipid metabolism; SEC: Membrane transport. MET: Metabolism of cofactors and vitamins. OAA: Metabolism of other aminoacids. NUC: Nucleotide metabolism. REP: Replication and repair. TRANSD: Transduction. TRANSC: Transcription. TRANSL: Translation. XEN: Xenobiotics biodegradation and metabolism. Significance was evaluated by means of a Kruska-Wallis and Mann-Whitney test, and plotted above bars when the test was positive.
Codes: 0 '***' 0.001 '**' 0.01 '*' 0.05 '.' 0.1 '' 1
