# Supplementary material for: Relating genomic characteristics to environmental preferences and ubiquity in different microbial taxa
Source: BMC Genomics. 2017 Jun 29;18:499. doi: 10.1186/s12864-017-3888-y (PMC5492924; doi:10.1186/s12864-017-3888-y)
Supplement: Supplementary file 5 — Linear regressions between COG ratios and genome size, for genera with different environmental preferences. Only significant instances (p-value <0.01) are shown. (PDF 199 kb) [file 12864_2017_3888_MOESM5_ESM.pdf]

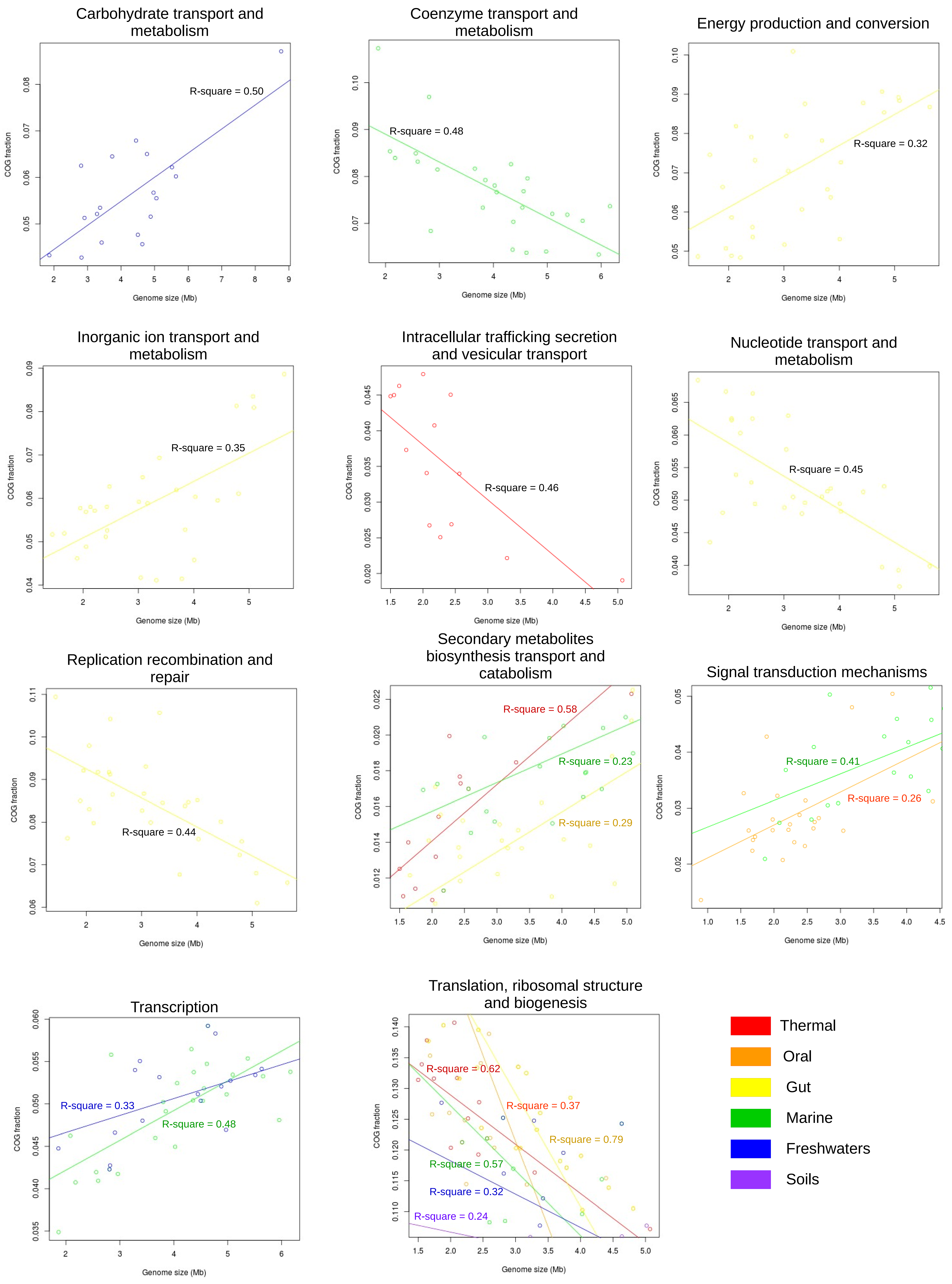
Carbohydrate transport and metabolism
R-square = 0.50
Coenzyme transport and metabolism
R-square = 0.48
Energy production and conversion
R-square = 0.32
Inorganic ion transport and metabolism
R-square = 0.35
Intracellular trafficking secretion and vesicular transport
R-square = 0.46
Nucleotide transport and metabolism
R-square = 0.45
Replication recombination and repair
R-square = 0.44
Secondary metabolites biosynthesis transport and catabolism
R-square = 0.58
R-square = 0.23
R-square = 0.29
Signal transduction mechanisms
R-square = 0.41
R-square = 0.26
Transcription
R-square = 0.33
R-square = 0.48
Translation, ribosomal structure and biogenesis
R-square = 0.62
R-square = 0.37
R-square = 0.79
R-square = 0.57
R-square = 0.32
R-square = 0.24
COG fraction
Genome size (Mb)
Thermal
Oral
Gut
Marine
Freshwaters
Soils
