# Supplementary material for: Relating genomic characteristics to environmental preferences and ubiquity in different microbial taxa
Source: BMC Genomics. 2017 Jun 29;18:499. doi: 10.1186/s12864-017-3888-y (PMC5492924; doi:10.1186/s12864-017-3888-y)
Supplement: Supplementary file 6 — Linear regressions between ORF ratio and genome size for genera with different environmental preferences. Only significant instances (p-value <0.01) are shown. (PDF 176 kb) [file 12864_2017_3888_MOESM6_ESM.pdf]

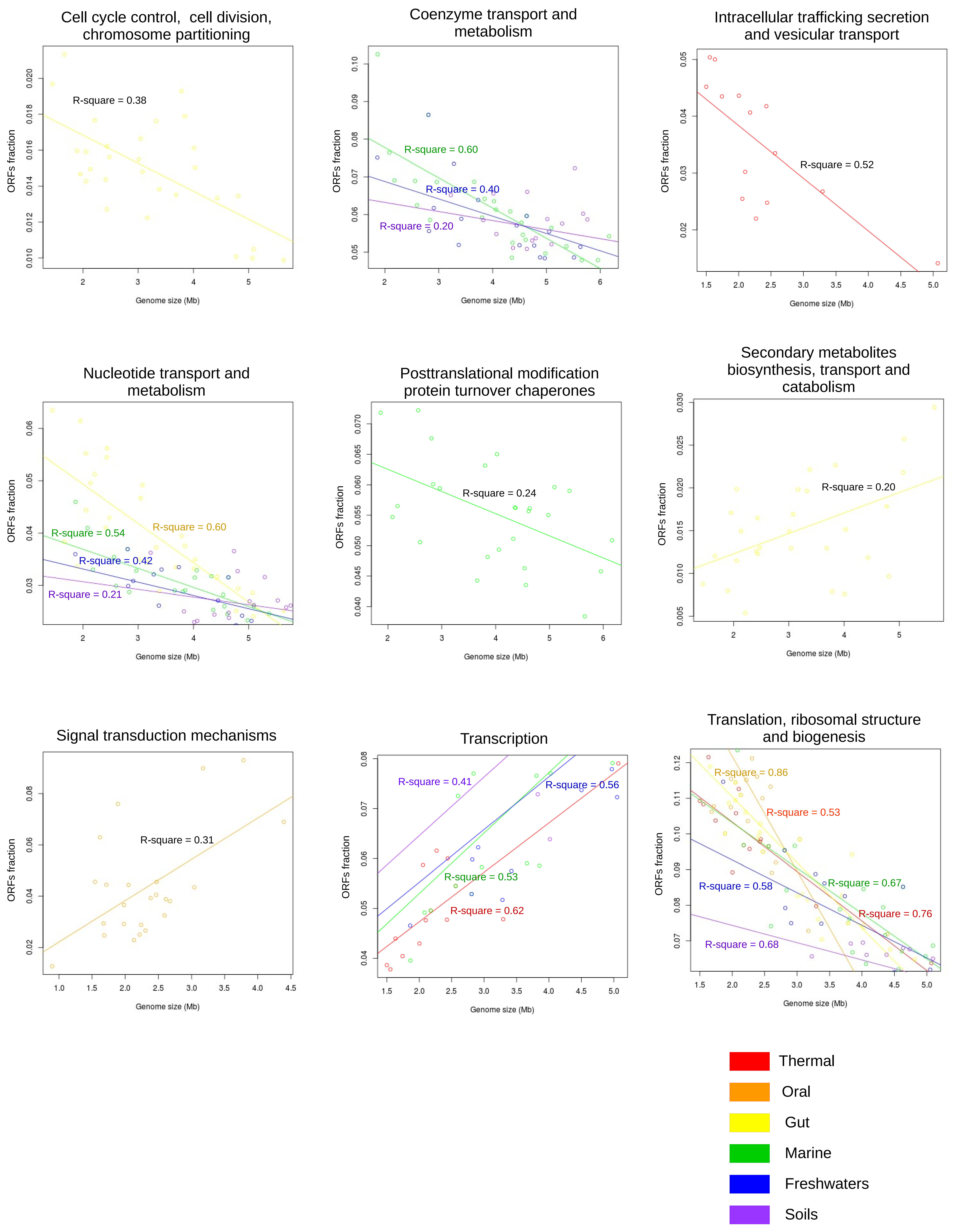

Cell cycle control, cell division, chromosome partitioning
R-square = 0.38
Coenzyme transport and metabolism
R-square = 0.60
R-square = 0.40
R-square = 0.20
Intracellular trafficking secretion and vesicular transport
R-square = 0.52
Nucleotide transport and metabolism
R-square = 0.60
R-square = 0.54
R-square = 0.42
R-square = 0.21
Posttranslational modification protein turnover chaperones
R-square = 0.24
Secondary metabolites biosynthesis, transport and catabolism
R-square = 0.20
Signal transduction mechanisms
R-square = 0.31
Transcription
R-square = 0.41
R-square = 0.56
R-square = 0.53
R-square = 0.62
Translation, ribosomal structure and biogenesis
R-square = 0.86
R-square = 0.53
R-square = 0.58
R-square = 0.67
R-square = 0.76
R-square = 0.68
ORFs fraction
Genome size (Mb)
Thermal
Oral
Gut
Marine
Freshwaters
Soils
